# Pink hypopyon in a patient with *Serratia marcescens* corneal ulceration

**DOI:** 10.1186/s12348-015-0041-4

**Published:** 2015-03-31

**Authors:** James A Stefater, Durga S Borkar, James Chodosh

**Affiliations:** Department of Ophthalmology, Massachusetts Eye and Ear Infirmary, Harvard Medical School, 243 Charles Street, Boston, MA 02114 USA

**Keywords:** Corneal ulcer, Pink hypopyon, *Serratia marcescens*

## Abstract

A 65-year-old woman presented to the emergency ward at the Massachusetts Eye and Ear Infirmary with 2 days of redness, irritation, photophobia, and diminished vision in her left eye. She was found to have a large central corneal ulcer with a small hypopyon. On the following day, after initiation of broad-spectrum antibiotics, the patient had improved symptoms but now had a 2-mm hypopyon that was distinctly pink in color. Cultures were positive for *Serratia marcescens.* A pink hypopyon, a rare occurrence, alerted the authors to a causative agent of Enterobacteriacae, either *Klebsiella* or *Serratia.* Immediate and intensive treatment was subsequently initiated.

A 65-year-old woman presented to the emergency ward at the Massachusetts Eye and Ear Infirmary with 2 days of redness, irritation, photophobia, and diminished vision in her left eye. She wore soft contact lenses but noted that she had not worn her lenses for the last 3 days. She also described a prior history of ocular herpes simplex virus in both eyes which last flared 7 months prior to her presentation. She noted compliance with her prophylactic oral acyclovir 400 mg twice daily. Her other medical problems include lumbar disk herniation, hypertension, gastroesophageal reflux disease, and cirrhosis secondary to alcohol use.

On exam, the patient was found to have normal vital signs. Her best corrected visual acuities were 20/40 in the right eye and 20/300 in the left eye. Anterior examination of her right eye was unremarkable except for a mild nuclear sclerotic and cortical cataract. On the left, she was noted to have erythema and tenderness of her eyelids with 3+ diffuse conjunctival injection. She had a soft contact lens in place in the left eye. The patient did not know there was a contact lens retained in her left eye. Slit lamp biomicroscopy of the left eye revealed diffuse corneal edema with a large pericentral epithelial defect (5.2 × 2.7 mm) with three small focal areas of infiltration measuring less than 1 mm, 4+ cell and flare, and a 1-mm hypopyon. The rest of the exam was unremarkable. At this time, the patient was presumed to have contact lens-related keratitis with concern for herpes simplex virus reactivation. A corneal scraping was performed for gram and calcofluor white stains and cultures, and the contact lens was also cultured. The patient was started on acyclovir 400 mg PO five times daily, cyclopentolate three times daily, fortified vancomycin 25 mg/ml every hour, and tobramycin 14 mg/ml every hour.

Upon return the following day, the patient was found to have improved symptoms but unchanged visual acuity. Exam revealed an improving epithelial defect and diffuse corneal stromal haze with moderate folds in Descemet’s membrane. The patient now had a 2-mm hypopyon that was distinctly pink in color (Figure 1). Cultures were subsequently positive for *Serratia marcescens* from both the corneal scrapings and the retained contact lens.

**Figure 1:**
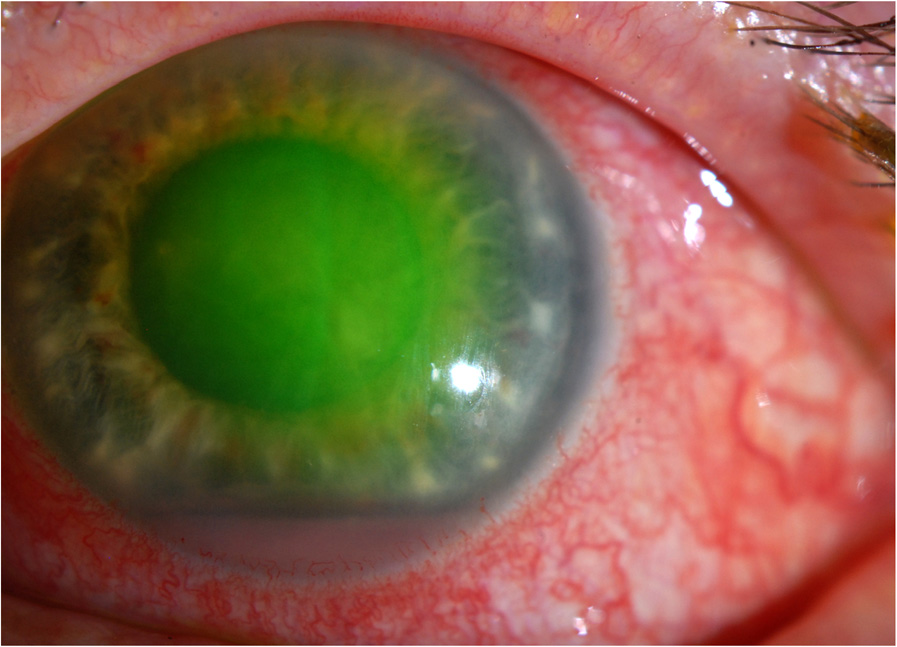
**The patient’s left eye demonstrating a pink hypopyon in the presence of fluorescein staining.**

In a recent report, *S. marcescens* makes up about 10% of culture isolates in bacterial keratitis [1]. Initially, we presumed that the pink nature of the hypopyon was secondary to coincident red blood cells with the leukocyte infiltrate. However, several reports have demonstrated an association between certain bacterial species and a specific color of intraocular infiltration. A pink hypopyon was first reported as a sign of *S. marcescens* endophthalmitis [2]. A subsequent report also identified *Klebsiella pneumonia* endophthalmitis as a cause of pink hypopyon [3]. Interestingly, the *S. marcescens* bacterium has been known for many years to produce a red pigment (prodigiosin). The red pigment has been identified in tooth abscesses and in the urine of patients with urinary tract infections [3,4].

In this report, we describe a case of a pink hypopyon caused by a contact lens-related corneal ulcer. Our patient responded well to immediate and intensive treatment with fortified aminoglycosides. A pink hypopyon should alert the physician to a causative agent that includes Enterobacteriacae, either *Klebsiella* or *Serratia,* and can occur in relation to contact lens over-wear*.*

## Consent

Written informed consent was obtained from the patient for the publication of this report and the accompanying image.
